# Benefits of an expanded use of plasma exchange for anti-neutrophil cytoplasmic antibody-associated vasculitis within a dedicated clinical service

**DOI:** 10.1186/s12891-015-0796-7

**Published:** 2015-11-09

**Authors:** Neeraj Dhaun, Andrew Saunders, Christopher O. Bellamy, Rocío Martinez Gallardo, Lynn Manson, David C. Kluth

**Affiliations:** BHF Centre of Research Excellence, University of Edinburgh, The Queen’s Medical Research Institute, 47 Little France Crescent, EH16 4TJ Edinburgh, UK; Department of Renal Medicine, Royal Infirmary of Edinburgh, Edinburgh, UK; Department of Pathology, Royal Infirmary of Edinburgh, Edinburgh, UK; Scottish National Blood Transfusion Service, Edinburgh, UK

**Keywords:** ANCA vasculitis, Outcome, Plasma exchange

## Abstract

**Background:**

Current recommendations for ANCA-associated vasculitis (AAV) support its management within a dedicated clinical service. Therapies for AAV are imperfect with many patients failing to achieve disease control and others experiencing disease relapse. Plasma exchange (PEX) may be beneficial especially when the kidney is involved.

**Methods:**

Within a new, dedicated service we retrospectively assessed, over a 6-year period, the benefits of PEX in two patient cohorts, discriminated by PEX treatment alone. Patients received PEX alongside standard of care if they fulfilled any of the following criteria: *1.* serum creatinine >500 μmol/l or dialysis-requiring renal failure, *2.* alveolar haemorrhage, *3*. renal biopsy showing ≥30 % focal and necrotising lesions ± cellular crescents. Outcome measures included disease remission and relapse, cumulative immunosuppression, and morbidity and mortality.

**Results:**

Of 104 new patients, 58 patients received PEX at presentation, 46 did not. Cyclophosphamide and/or rituximab dosing was similar for both groups. Although patients receiving PEX had poorer renal function, a higher C-reactive protein and disease activity score at presentation disease remission rate was similar in both groups (no PEX *vs.* PEX: 96 % *vs.* 98 %). The PEX group entered remission quicker (no PEX *vs.* PEX: 3.9 ± 4.0 *vs.* 2.8 ± 1.3 months, *p* < 0.05), with a lower 3-month cumulative glucocorticoid dose (no PEX *vs.* PEX: 2.5 ± 0.4 *vs.* 2.3 ± 0.2 g, *p* < 0.001). Relapse was similar between groups but adverse events lower in the PEX group.

**Conclusions:**

PEX may be of benefit in AAV. Larger, longer randomised controlled trials are now needed.

**Electronic supplementary material:**

The online version of this article (doi:10.1186/s12891-015-0796-7) contains supplementary material, which is available to authorized users.

## Background

Systemic vasculitis associated with autoantibodies to neutrophil cytoplasmic antigens (ANCA) encompasses granulomatosis with polyangiitis (GPA, previously known as Wegener’s granulomatosis), microscopic polyangiitis (MPA) and eosinophilic granulomatosis with polyangiitis (EGPA, previously known as Churg-Strauss syndrome). ANCA-associated systemic vasculitis (AAV) has a prevalence of 14–30 patients per 100,000 [1] and its most frequent severe manifestations include rapidly progressive glomerulonephritis leading to dialysis-requiring renal failure and alveolar capillaritis leading to pulmonary haemorrhage. Given its rare nature current guidelines recommend referral of all patients with AAV to a specialised service [2]. In line with this we designed and set up a new clinical service in our centre with the aim of centralising expertise to improve patients outcomes, and to facilitate patient participation in clinical trials of AAV.

Current standard of care for the initial treatment of AAV is glucocorticoids in combination with either cyclophosphamide (CYC) or the B cell depleting antibody, rituximab. Despite treatment, however, overall survival in AAV remains poor with many patients suffering chronic morbidity including end-stage renal disease [3, 4]. These are considered to be as a result of ineffective therapy and treatment toxicity.[4] At least 20 % of AAV patients do not achieve adequate disease control and an additional ~50 % relapse within 5 years [5, 6]. These both result in increased immunosuppressive burden with its associated risks, most importantly infection [7]. Between 25 and 50 % of patients with severe AAV have a severe infection within 12 months of starting treatment and the most common causes of death are infection and uncontrolled disease activity [8, 9]. Thus, there is an unmet need for additional therapies that not only improve disease control but also limit treatment toxicity.

Plasma exchange (PEX) removes circulating plasma constituents including immunoglobulins. Its potential role in the treatment of AAV has been proposed since the discovery of the pathogenic role of ANCA in AAV [10, 11]. Data from small studies suggest that PEX may be of benefit in AAV [12, 13]. Current recommendations for the use of PEX in AAV include pulmonary haemorrhage [14] and severe renal disease [15]. The latter is defined as either a serum creatinine >500 μmol/l or dialysis-dependent renal failure but this is largely based on the MEPEX study [8] which compared PEX with intravenous methylprednisolone as adjuvants to CYC and oral prednisolone in patients with a new diagnosis of AAV and severe renal disease.

However, measurement of renal function, using serum creatinine is inadequate as substantial irreversible renal tissue damage can occur before function is impaired to a detectable extent [16]. Histological features of renal injury in AAV include an intense, neutrophil-predominant inflammatory infiltrate, segmental glomerular necrosis reflecting a glomerular capillaritis and intra-glomerular monocyte proliferation contributing to a pauci-immune, focal and necrotising, crescentic glomerulonephritis [17]. In this first study from our dedicated vasculitis service, we compared the addition of PEX on top of standard of care to standard of care alone in patients presenting with a new diagnosis of AAV. Indications for PEX were either pulmonary haemorrhage or ‘severe’ renal disease. Importantly, alongside the standard dialysis-requiring renal failure and serum creatinine criteria we included a histological definition of severity. Thus, ‘histologically significant renal disease’ was also defined as ≥30 % focal and necrotising lesions with or without cellular crescents on renal biopsy. Our outcome measures included disease remission and relapse, morbidity and mortality.

## Methods

### Design & set-up of a new vasculitis service

All patients with AAV presenting to our unit and those referred from others were managed in the new specialised vasculitis service covering NHS Lothian & Borders, a population of ~1 million. The components of this service included:

#### Clinical personnel

Two renal consultants; a single, dedicated renal pathologist to review all renal vasculitis biopsies so minimising variation in histological data interpretation; two radiologists able to perform renal and/or lung biopsies within 24–48 h of patient presentation; a supportive plasma exchange service headed by an interested haematologist; a core group of nurses trained to administer biological agents and intravenous CYC.

#### Clinical assessments

All inpatients with AAV were reviewed by the vasculitis team daily; on discharge they were reviewed in the dedicated clinic fortnightly for the first 6 weeks, and then monthly for the first 6 months; thereafter, clinic attendance was 3-monthly or earlier if needed (suggestion of disease relapse or disease complications).

#### Patient support

All AAV patients were given a central email address and mobile number that they could call or text if they experienced any problems with symptoms or their treatment. Queries were responded to within 24 h. Patients were also given the contact details for a national vasculitis patient support group ‘The Lauren Currie Twilight Foundation’.

See: http://www.thelaurencurrietwilightfoundation.org/

#### Broader education

To improve general awareness of AAV and of the new specialised clinical service the two clinicians responsible for the service gave talks to primary and secondary care as well as at the patient support group.

#### Succession planning

Interested trainees from nephrology, neurology, rheumatology and respiratory medicine were invited to undertake 6-month attachments in the vasculitis service: they attended clinics, organised treatments and answered patient queries under the guidance of the lead clinicians.

### Study comparing PEX vs. no PEX

#### Patient population

All patients presenting to the new dedicated service between September 2006 and April 2013 were included in the study. Patients were categorised by ANCA status and extent of organ involvement and followed up for the time period of the study. Patients positive for both ANCA and anti-glomerular basement membrane antibody were excluded from the analysis. As the data presented are a retrospective analysis of routine clinical care, the South-East Scotland Local Research Ethics Committee advised that this study did not require ethical approval or patient consent.

### Clinical data

Data collection included demographic characteristics, disease activity and damage assessments, medications, and laboratory results at each assessment and severe adverse events since starting treatment. Disease activity was graded according to the ‘Birmingham Vasculitis Activity Score’ (BVAS, scores range from 0 to 63, with higher scores indicating more active disease) [18] and by investigators’ assessments of disease activity as remission, ongoing active disease (treatment failure), or relapse. Remission was defined as a BVAS score of 0 that was maintained for 2 months and a prednisolone dosage of ≤10 mg/day. Treatment failure occurred when remission was not achieved and disease activity progressed, necessitating additional immunosuppression. Disease relapse was defined as vital organ-threatening vasculitis activity, such as, disease of the eyes, lungs, kidneys, or sub-glottis, or other manifestations attributable to active vasculitis necessitating escalation of immunosuppression. Damage related to disease or treatment was scored according to the ‘Vasculitis Damage Index’ (VDI, scores for this index range from 0 to 64, with higher scores indicating more severe damage) [19]. Severe adverse events were categorised as those resulting in hospitalisations, serious infections – defined as those requiring hospitalisation for intravenous therapy – malignancies, or death.

### Treatment protocols

#### Plasma exchange (PEX)

All patients were allocated to treatment with PEX alongside standard of care if they fulfilled any of the following criteria at presentation: *1.* serum creatinine >500 μmol/l or dialysis-requiring renal failure, *2.* alveolar haemorrhage, *3*. significant histological renal disease defined as ≥30 % focal and necrotising lesions ± cellular crescents on renal biopsy (see Additional file 1: Supplementary Methods for further information on renal biopsies). PEX consisted of 8 centrifugal exchanges (5 initially given on consecutive days and then 3 on alternate days). Each exchange comprised of 60 ml/kg with 4 % human albumin solution used as the replacement fluid. In patients at risk of bleeding fresh frozen plasma was used as the replacement. All patients receiving PEX were prescribed supplemental oral calcium (~2400 mg/day) and cholecalciferol (~40 μg/day).

#### Glucocorticoids

Oral glucoroticoids were given at a starting dose of 1 mg/kg/day to a maximum dose of 80 mg/day. The dose reduction schedule is shown in the Additional file 1: Supplementary Methods. Pulsed steroids were not used.

#### Cyclophosphamide (CYC)

Between 2006 and 2007 CYC was given orally at a dose of 2 mg/kg/day to a maximum dose of 150 mg/day. From 2007 onwards, intravenous (i.v.) CYC was used with the dose adjusted for age and renal function (see Additional file 1: Supplementary Methods).

#### Rituximab

Rituximab was given as 2 i.v. doses of 1 g given two weeks apart. Premedication comprised of hydrocortisone 200 mg i.v, paracetamol 1 g oral, and chlorpheniramine 10 mg i.v.

#### Timing of CYC & rituximab

The first dose of i.v. CYC and/or rituximab was given on completion of the 5 initial consecutive day PEX and there was at least 24 h delay between the completion of either CYC or rituximab and the next PEX.

#### Mycophenolate mofetil (MMF)

This was prescribed at a dose of 1 g twice a day (b.d.).

#### Methotrexate

This was given orally at a dose of 5 – 20 mg/week with co-prescription of folic acid.

### Study endpoints

These included disease remission and relapse, time to relapse, cumulative immunosuppressive burden at 3 months, severe adverse events and all-cause mortality.

### Statistical analysis

Statistical analysis was performed using SPSS version 18 and Prism version 6 software. Results are expressed as actual values and percentages for categorical variables and as means ± standard deviations or median ± interquartile range for continuous variables. Group comparisons were made using Fisher’s exact test, student *t*-test, Wilcoxon-Mann–Whitney and one-way analysis of variance (with repeated measures) where appropriate. Relapses were analysed using Kaplan-Meier survival analysis, with log rank analysis for significance. *P* values less than 0.05 were considered significant.

## Results

### Specialised Vasculitis Service (Table 1)

**Table 1:** Demographic data, clinical parameters at presentation, treatments given, and outcome data for all patients within the vasculitis service and also categorised by ANCA subtype

Characteristic	PR3+ (*n* = 51)	MPO+ (*n* = 46)	ANCA-^a^ (*n* = 7)	Overall (*n* = 104)
Age	58 ± 15	64 ± 19	61 ± 4	61 ± 16
Male / Female	26 /25	21 / 25	5 / 2	52 / 52
Renal involvement (%)	45 (88)	44 (96)	4 (57)	93 (89)
At presentation				
Creatinine (μmol/l)	225 ± 225	330 ± 239	234 ± 216	272 ± 235
CRP (mg/l)	97 ± 86	73 ± 73	38 ± 40	82 ± 79
Haemoglobin (g/l)	107 ± 23	98 ± 18	114 ± 28	103 ± 22
Dialysis-requiring	9	11	1	21
Induction period (~3 months)				
Number (%) of patients receiving				
Glucocorticoids	51 (100)	46 (100)	7 (100)	104 (100)
CYC	35 (69)	30 (65)	3 (43)	68 (65)
MMF	9 (18)	18 (39)	2 (29)	29 (28)
Rituximab	6 (12)	15 (33)	1 (14)	22 (21)
Plasma exchange	25 (49)	30 (65)	3 (43)	58 (56)
Cumulative glucocorticoid dose (g)	2.3 ± 0.3	2.4 ± 0.3	2.2 ± 0.0	2.3 ± 0.3
Cumulative CYC dose (g)	6.5 ± 3.2	6.4 ± 3.9	5.3 ± 1.0	6.3 ± 3.4
Remission				
Number entering remission (%)	50 (98)	44 (96)	7 (100)	101^b^ (97)
Time to remission (days)	108 ± 184	82 ± 29	124 ± 149	98 ± 136
At 12 months				
Creatinine (μmol/l)	124 ± 57	215 ± 193	120 ± 68	158 ± 132
Dialysis-requiring	1	4	0	5
Relapses & mortality				
Number of patients with disease relapses (%)	15 (29)	4 (9)	1 (14)	20 (19)
Mean time to first relapse (days)	880 ± 692	1073 ± 877	635 ± 760	953 ± 786
Median time to first relapse (days & IQR)	754 (823)	857 (1252)	223 (1078)	795 (1102)
Number of patients died (%)	5 (10)	4 (9)	0 (0)	9 (9)

*ANCA* anti-neutrophil cytoplasmic antibody, *BVAS* Birmingham Vasculitis Activity Score, *EGPA* eosinophilic granulomatosis with polyangiitis, *ENT* ear, nose & throat, *GPA* granulomatosis with polyangiitis, *MPA* microscopic polyangiitis, *MPO* myeloperoxidase, *PR3* proteinase-3. Data are shown as number of patients (%), mean ± standard deviation or median and IQR. ^a^The 7 patients that were ANCA- had evidence of a pauci-immune necrotising glomerulonephritis on renal biopsy. ^b^3 patients did not enter remission as they died during before this

**Fig. 1:**
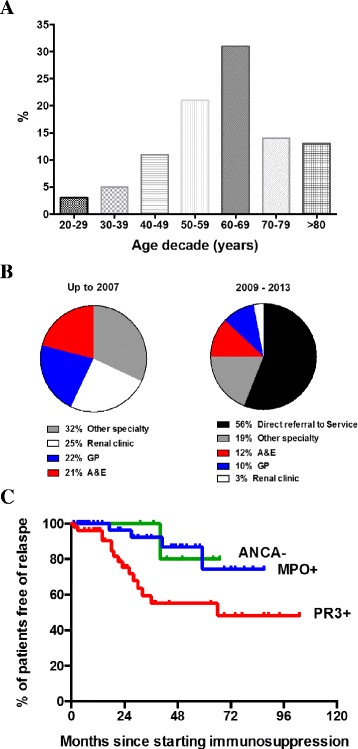
Age distribution, source of referral and relapse-free survival curves for all study patients. **a** age distribution at presentation of all patients cared for within the vasculitis service. Data are shown as % frequency within each 10-year period. **b** source of referral of vasculitis patients up to 2007 (left) and 2009 – 2013 (right). **c** relapse-free survival curves for patients in the groups defined by their ANCA status at presentation (PR3: proteinase-3, red line, *n* = 51; MPO: myeloperoxidase, blue line, *n* = 46; ANCA-: ANCA negative, green line, *n* = 7). *p* = 0.02 for PR3 *vs.* MPO and for PR3 *vs.* ANCA- by log rank analysis

Demographic data for the overall group of patients managed within the vasculitis clinic and classified by ANCA status are shown in Table 1. The age distribution of patients at disease presentation is shown in Fig. 1a. 29 patients (28 %) of all incident patients were ≥70 years of age. When comparing groups by ANCA status, age was similar as was the number of male and female patients.

Over the study period the source of patient referral changed. Up to 2007 incident and prevalent patients with vasculitis had a similar referral pattern: from another medical specialty, the general nephrology clinic, primary care and the emergency department. Once the vasculitis service had been established for 5 years this changed with most incident patients referred directly to the service (Fig. 1b). Over the same time periods, the mean time to renal biopsy changed from 4.6 to 2.3 days and the median from 2.0 days to 1.0.

Mean and median follow up of all patients were 1001 ± 860 and 772 ± 86 days, respectively. When categorised by ANCA status, remission rates and time to remission were similar across groups. In terms of disease relapse, those with disease associated with PR3 ANCA were more likely to relapse (PR3 *vs.* MPO *vs.* ANCA-: 29 *vs.* 9 *vs.* 14 %, *p* = 0.02 by log rank analysis for both PR3 *vs.* MPO and PR3 *vs.* ANCA-, Fig. 1c).

Outcomes were no different between those patients who received CYC (n = 68) as part of their initial induction compared to those who received rituximab (*n* = 22) (See Additional file 1: Table S1).

Of the 29 patients who were ≥70 years at presentation 27 were alive at 1 year after the start of treatment (See Additional file 1: Table S2). Elderly patients were more likely to be treated with rituximab than younger patients (age ≥70 *vs.* <70: 48 *vs.* 11 %, *p* < 0.0001).

### PEX vs. no PEX

Within the time period of the study 104 patients presented with vasculitis. Of these, 58 patients received PEX as part of their induction treatment, 46 did not. Indications for PEX at presentation were dialysis-dependent renal failure, *n* = 20 (35 %); creatinine >500 μmol/l, *n* = 12 (21 %); alveolar haemorrhage, *n* = 9 (16 %); and significant histological renal disease, *n* = 32 (55 %).

Demographics of the two patient groups are shown in Table 2. There were no differences between the two groups in terms of age, sex distribution, or ANCA status. Extent of organ involvement was also similar apart from nerve disease, which was commoner in those that did not receive PEX. BVAS was higher in those receiving PEX. Mean follow up was 24.1 ± 21.5 months in the group of patients receiving PEX and 43.2 ± 32.3 months in those not receiving it. Follow up >5 years was available for 6 patients who received PEX and 17 who did not.

**Table 2:** Baseline demographic data of the groups either receiving or not receiving plasma exchange (PEX) as part of disease induction

Characteristic	No PEX (*n* = 46)	PEX (*n* = 58)	*p* value
Age (years)	61 ± 15	60 ± 17	0.81
Male / Female	26 / 20	27 / 31	-
ANCA status (%)			
PR3+	26 (57)	25 (43)	0.67
MPO+	16 (35)	30 (52)	0.07
Negative	4 (8)	3 (5)	0.81
Diagnosis			
GPA	26	25	0.67
MPA	12	29	0.02
ANCA- vasculitis	4	3	0.81
EGPA	3	1	0.32
Organ involvement			
Number (%) of patients			
Kidney	35 (76)	57 (98)	0.47
Lung	26 (46)	30 (52)	0.54
ENT	17 (37)	13 (22)	0.89
Nerve	14 (30)	8 (14)	0.03
Skin	10 (22)	8 (14)	0.26
Joints	7 (15)	11 (19)	0.65
Eye	4 (9)	5 (9)	0.96
Gastrointestinal	1 (2)	3 (5)	0.44
Indication for PEX			
Number (%) of patients			
Dialysis-requiring renal failure	-	20 (34)	
Serum creatinine ≥500 μmol/l	-	12 (21)^a^	
Alveolar haemorrhage	-	9 (16)	
Histologically significant renal disease	-	52 (90)	
BVAS	17 (13 – 23)	29 (12 – 47)	0.01

Abbreviations are as for Table 1. Data are given as number of patients (%) with median and interquartile range shown for BVAS. ^a^10 of these 12 patients required dialysis at disease presentation

### Disease remission

**Table 3:** Clinical parameters at presentation, treatments given, and outcome data for the two groups either receiving or not receiving plasma exchange (PEX) as part of disease induction

Characteristic	No PEX (*n* = 46)	PEX (*n* = 58)	*p* value
At presentation			
Creatinine (μmol/l)	140 ± 90	370 ± 259	0.000
eGFR (ml/min/1.73 m^2^)	56 ± 31	22 ± 22	0.000
CRP (mg/l)	48 ± 61	105 ± 82	0.001
Haemoglobin (g/l)	116 ± 21	94 ± 18	0.000
Albumin (g/l)	34 ± 6	29 ± 6	0.000
Dialysis-requiring	1^a^	20	0.000
VDI	0	0	1
Induction period (~3 months)			
Number (%) of patients receiving			
Glucocorticoids	46 (100)	58 (100)	1
CYC	23 (50)	45 (78)	0.004
Rituximab	15 (33)	7 (12)	0.03
CYC *and/or* rituximab	38 (83)	52 (90)	0.39
MMF	8 (17)	16 (28)	0.24
Methotrexate	8	0	0.001
Azathioprine	6	1	0.02
Infliximab	1	0	0.25
Cumulative glucocorticoid dose (g)	2.5 ± 0.4	2.3 ± 0.2	0.000
Cumulative CYC dose (g)	8.0 ± 3.6	5.4 ± 3.0	0.002
Disease remission			
Number (%) entering remission	44 (96)	57 (98)	0.98
Time to remission (days)	118 ± 124	83 ± 39	0.046
At 12 months			
Creatinine (μmol/l)	120 ± 46	191 ± 172	0.02
eGFR (ml/min/1.73 m^2^)	56 ± 24	43 ± 24	0.02
CRP (mg/l)	22 ± 71	8 ± 14	0.33
Haemoglobin (g/l)	126 ± 14	121 ± 15	0.15
Albumin (g/l)	41 ± 6	40 ± 5	0.31
Dialysis-requiring	0	5	0.04
VDI	1.2 ± 0.2	1.3 ± 0.1	0.79
Disease relapse			
Number (%) of patients with relapses	12 (26)	8 (14)	0.09
Mean time to first relapse (days)	714 ± 699	564 ± 318	0.38
Median time to first relapse	800 (1124)	773 (1164)	0.57
(days & IQR)			

*CRP* C-reactive protein, *CYC* cyclophosphamide, *eGFR* estimated glomerular filtration rate, *IQR* interquartile range, *MMF* mycophenolate mofetil, *VDI* Vasculitis Damage Index. Data are shown as either mean ± standard deviation or median and IQR. ^a^This patient did not receive PEX as it was not tolerated alongside dialysis

**Fig. 2:**
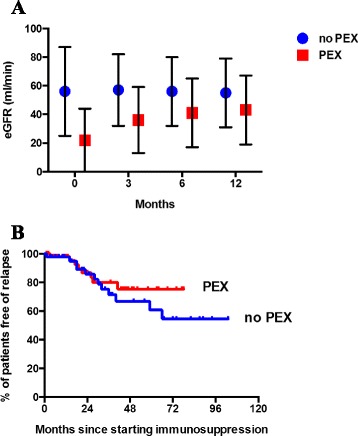
Change in eGFR and relapse-free survival curves for PEX vs. no PEX patients. **a** change in abbreviated 4-variable MDRD estimated glomerular filtration rate (eGFR) over the 12 months since starting treatment. Data are shown as mean ± standard deviation. Blue circles: received no plasma exchange (PEX); red squares: received PEX. At each time point eGFR was different between the two groups (*p* < 0.05) based on a repeated measures ANOVA. Between 0 and 12 months there was no change in eGFR in the no PEX group whereas eGFR improved in the PEX group (*p* < 0.001). **b** relapse-free survival curves for patients in the group receiving plasma exchange (PEX, red line, *n* = 58) up to 6 years and for those not receiving it (blue line, *n* = 46) up to 10 years. *p* = 0.64 by log rank analysis

Table 3 shows the outcome data for the two groups. Despite the group of patients receiving PEX being sicker at presentation (as illustrated by poorer renal function, a greater inflammatory response and higher disease activity score) disease remission rate was similarly high to the group that did not receive PEX (no PEX *vs.* PEX: 96 % *vs.* 98 %, *p* = ns). Interestingly, those receiving PEX entered remission quicker than those who did not (no PEX *vs.* PEX: 3.9 ± 4.0 *vs.* 2.8 ± 1.3 months, *p* < 0.05). In those patients receiving PEX, estimated GFR (eGFR) improved over the 12 months following start of treatment (0 *vs.*12 months: 22 ± 22 *vs.* 43 ± 24 ml/min, *p* < 0.001), whereas it did not change in the group not receiving PEX (0 *vs.*12 months: 56 ± 31 *vs.* 55 ± 24 ml/min, *p* = ns, Fig. 2a).

At 12 months C-reactive protein, haemoglobin and serum albumin were normal in both groups and did not differ between the groups. Of the 20 patients requiring dialysis at presentation in the PEX group only 5 remained dialysis-dependent at 12 months. The one patient requiring dialysis in the no PEX group also regained independent renal function at 12 months.

### Cumulative immunosuppression (Table 3)

All 104 patients received glucocorticoids at induction but the cumulative dose at 3 months was lower in those who also received PEX than in those that did not (no PEX *vs.* PEX: 2.5 ± 0.4 *vs.* 2.3 ± 0.2 g, *p* < 0.001). ~80 % of patients who were treated with PEX were also treated with CYC, whereas CYC was the induction immunosuppression of choice in ~50 % of the non-PEX group. Cumulative CYC dose was ~30 % lower in the PEX group than in the non-PEX group (*p* < 0.01), likely related to poorer renal function in the PEX group. Rituximab use was commoner in the group not receiving PEX (33 *vs.* 12 %), although overall, both groups did not differ in whether they received CYC *and/or* rituximab.

### Disease relapse (Table 3)

Relapse rates were similar between the groups as was the time to first relapse (no PEX *vs.* PEX: 26 *vs.* 14 % and 23.8 ± 13.3 *vs.* 18.8 ± 10.6 months, *p* = ns for both, Fig. 2b).

### Adverse events

**Table 4:** Serious adverse events for the groups either receiving or not receiving plasma exchange (PEX) as part of disease induction. Data are given as number of patients (%)

Severe adverse events Number (%)	No PEX (*n* = 46)	PEX (*n* = 58)	*p* value
All severe adverse events			
Number of adverse events	21	15	0.04
Number (%) of patients	15 (33)	12 (21)	0.26
Serious infections, number of events	10	7	0.29
Chest	6	5	0.53
Urine	2	2	1
*Pneumocystis jirovecii* pneumonia	2	0	0.19
Other hospitalizations, number of events	4	5	1
Fractures	2	2	1
Cardiovascular	2	3	1
Malignancy	2	0	0.19
Death	5	3	0.46

 Table 4 shows the adverse events for the two groups. Of the 58 patients receiving PEX, 3 experienced symptomatic hypocalcaemia – mean corrected serum calcium was 1.96 ± 0.14 mmol/l. Although PEX was started at least 24 h following a renal biopsy 2 patients had significant post-biopsy bleeds that required blood transfusion. There were no episodes of central venous cannula infection.

There were 15 serious adverse events in 12 of the 58 patients (21 %) receiving PEX and 21 events in 15 of the 46 patients (33 %) who did not receive PEX. The categorisation of these events is shown in Table 4. The total number of adverse events was lower in the PEX group (*p* = 0.04).

## Discussion

The evidence base for the treatment of AAV has been transformed over the last 15 years. Clinical trials have shown the effectiveness of cytotoxic drugs [20, 21], plasma exchange [8] and biological agents [9, 22]. However, clinical trials are often conducted in a controlled environment and results in everyday clinical practice can be very different [23]. Furthermore, many trials require stringent entry criteria to be met for subject inclusion and if these are rigidly applied to clinical practice it can often result in many patients missing out on potentially beneficial treatments. The current data represent our clinical experience in establishing a specialised service for the care of patients with AAV, which is a rare disease that has ‘orphan’ status in both the US and EU. The service involved a centralised clinic run by a small cohort of dedicated clinicians. Based on trial data and our own experience we developed local protocols for the management of AAV with the aim of optimising the clinical care and outcomes of patients with AAV. Our results suggest that those with AAV may benefit from PEX, with its expanded use based on histological criteria as well as the more conventional clinical and functional markers. PEX resulted in equivalent remission rates compared to non-PEX patients, despite those receiving PEX having more severe disease at presentation. Additionally, remission was achieved more rapidly and the cumulative steroid dose was lower. This latter finding likely explains the fewer adverse events in those receiving PEX.

The MEPEX trial compared PEX with intravenous methylprednisolone to the then standard of care in patients with AAV who were either dialysis-dependent or had a serum creatinine >500 μmol/l at presentation [8]. The main outcome of the study was that there was a reduced incidence of dialysis-dependent renal failure at 3 and 12 months, although no difference in mortality was observed. Interestingly, longer-term data from this study suggest that PEX confers no survival benefit [24]. MEPEX only included patients with very poor renal function. Furthermore, it used serum creatinine as its surrogate measure of GFR. This is a poor measure of renal function [16] and based on this alone MEPEX would have excluded many patients with a similar GFR to those included. This is an issue particularly in older patients [25]. Bearing this problem in mind we applied renal biopsy criteria to identify patients with ‘severe renal disease’ who might benefit from PEX. Although previous classifications have used ≥50 % glomeruli affected as a definition of severe renal disease this is arbitrary and prone to significant disease mis-classification depending on the adequacy of the renal biopsy. We defined ‘severe’ by the presence of ≥30 % focal and necrotising lesions in the presence or absence of cellular crescents on renal biopsy. In the MEPEX study, analysis of those patients who had renal biopsies suggested that ~80 % of glomeruli showed these histological features. Applying our broader criteria, 98 % of PEX patients achieved remission at a median interval of 2.8 months and eGFR improved from 22 ml/min at presentation to 44 ml/min at 12 months. Furthermore, 15 of the 20 dialysis-dependent patients were dialysis independent by 12 months. PEX was well tolerated with fewer adverse events compared to non-PEX treated patients despite more severe disease at presentation. Interestingly, our findings are similar to those of MEPEX with identical disease relapse rates.

There is significant evidence that ANCA are pathogenic [26]. They are able to activate neutrophils, causing production of reactive oxygen species and degranulation [27] and can cause endothelial injury [28]. Furthermore, animal models have shown that MPO antibodies and anti-MPO specific T-cells can induce a crescentic glomerulonephritis and lung disease [10, 29]. Thus, there is biological plausibility in believing that an early reduction in circulating ANCA titre may translate to clinical benefit. This may then partly explain why the cohort of patients receiving PEX achieved disease remission more rapidly. Unfortunately, we did not have a sufficient number of ANCA titres from the early treatment period to compare how they changed between the two groups. As with other studies [20, 28], we found distinct clinical phenotypes depending on the type of ANCA present. MPO positive patients presented with a higher serum creatinine and had worse renal function at 12 months, whereas PR3 positive disease was more likely to relapse [22, 30].

During the course of our study a number of clinical trials were published comparing the use of rituximab with cyclophosphamide for inducing disease remission [9, 22, 31]. Consistent with these data we found rituximab to be as effective as cyclophosphamide for the early treatment of disease (without an excess of adverse events) and so this study further supports the case for rituximab to be considered as standard of care for the initial treatment of AAV. This also fits with evidence that it is glucocorticoid dose during disease induction that is most linked to the increase in infection risk rather than the cytotoxic or biological agent is used [32].

AAV is increasingly identified in older patients, many of whom will have additional co-morbidities [33, 34]. In our population the annual incidence of AAV is ~58 cases per million of population in patients ≥70 years of age compared to ~20 per million in those <70. Our data also suggest that ~30 % of new cases of AAV occur in those >70 years. Overall, this age group represents ~12 % of our population. Our own practice has been to use rituximab in patients over the age of 70 alongside lower doses of glucocorticoids with the aim of reducing early infective complications [9, 31]. These patients had similar remission rates and 1 year survival compared to the younger cohort. Our data provide further evidence to support treatment with immunosuppression of older patients presenting with AAV [35], as well as the need to educate healthcare professionals about identifying disease in the elderly.

Providing a centralised clinical service for vasculitis has a number of inherent advantages. It allows the development of clinical expertise for a rare disease allowing prompt application of evidence based practice and will provide a forum for education of both patients and health professionals to facilitate more rapid specialist referral. Once the clinical service was established more than half the patients were directly referred to the vasculitis clinic. This resulted in more rapid diagnosis, appropriate investigation and initiation of treatment. The outcomes of our clinical service compare favourably with data from other vasculitis studies. We found that >95 % of our patients achieve disease remission by 3 months compared to 80-85 % of patients in most studies of either cyclophosphamide or rituximab. Major disease relapse occurred in 29 % of PR3 positive patients, 9 % MPO positive patients and 14 % of ANCA negative patients. These rates are lower than those from most published long-term clinical studies [5, 36]. This likely reflects the close monitoring of individual patients within a relatively small clinical service where interpretation of minor symptoms and clinical markers (urinalysis, C-reactive protein, serum creatinine, platelet count, ANCA titre) in identifies minor disease relapses leading to modifications of therapy that potentially prevent more major disease activity.

## Conclusions

Overall, our data show the benefits in developing a specialised clinical service for AAV, especially for the elderly population. Our disease remission and relapse rates, as well as adverse events, compare favourably with other published studies. Our modified use of PEX as part of disease induction therapy to incorporate those patients with histological evidence of severe renal disease suggests encouraging responses to treatment with no increase in adverse events.

### Limitations

We recognise the limitations of this work. Although the numbers of patients in our study are similar to those in previous published studies [9, 30, 37] the number is still reasonably low with likely inadequate statistical power to detect the benefits of PEX. Furthermore, this was a retrospective review and not a randomised clinical trial but the eagerly awaited results of the on-going PEXIVAS study [38], which extends PEX to patients with an eGFR <50 ml/min, should help to further clarify its role in the therapeutic armamentarium of AAV.

## Additional file

Additional file 1:
**Supplementary Methods.** **Table S1.** Comparison of patients receiving rituximab or cyclophosphamide (CYC) for induction. Data are shown as number of patients (%), mean ± standard deviation or median and IQR. **Table S2.** Comparison of rituximab dosing, cumulative glucocorticoid dose at 3 months, remission, relapse and survival in patients <70 and ≥70 years of age. Data are shown as number of patients (%), mean ± standard deviation or median and IQR. (DOCX 114 kb)
