# Supplementary material for: Benefits of an expanded use of plasma exchange for anti-neutrophil cytoplasmic antibody-associated vasculitis within a dedicated clinical service
Source: BMC Musculoskelet Disord. 2015 Nov 9;16:343. doi: 10.1186/s12891-015-0796-7 (PMC4640165; doi:10.1186/s12891-015-0796-7)
Supplement: Additional file 1: — Supplementary Methods. Table S1. Comparison of patients receiving rituximab or cyclophosphamide (CYC) for induction. Data are shown as number of patients (%), mean ± standard deviation or median and IQR. Table S2. Comparison of rituximab dosing, cumulative glucocorticoid dose at 3 months, remission, relapse and survival in patients <70 and ≥70 years of age. Data are shown as number of patients (%), mean ± standard deviation or median and IQR. (DOCX 114 kb) [file 12891_2015_796_MOESM1_ESM.docx]

**Supplementary Methods**

*Renal biopsies*

Renal biopsies were performed when there was a clinical suspicion of renal involvement – reduced renal excretory function or the presence of hematuria and/or proteinuria. They were carried out by a dedicated renal radiologist using a 14G biopsy needle and under ultrasound guidance. Tissue was collected for light microscopy, immunofluorescence and, where available, for electron microscopy. A dedicated renal pathologist evaluated all biopsies.

*Glucocorticoid dose reduction regimen*

Starting prednisone dose1mg/kg/day to a maximum of 80mg/day. Dose adjusted by clinicians as indicated.

| **Week** | **Starting prednisone dose (mg/day)** | **Starting prednisone dose (mg/day)** |
| --- | --- | --- |
| 1 | 60 | 80 |
| 2 | 45 | 60 |
| 3 | 30 | 45 |
| 4 | 25 | 30 |
| 5 | 20 | 25 |
| 6 | 20 | 20 |
| 7 | 17.5 | 20 |
| 8 | 17.5 | 17.5 |
| 9 | 15 | 17.5 |
| 10 | 15 | 15 |
| 11 | 12.5 | 15 |
| 12 | 12.5 | 12.5 |
| 13 | 10 | 12.5 |
| 14 | 10 | 10 |

*Cyclophosphamide dosing regimen*

Pulsed i.v. cyclophosphamide (CYC) was given 2 weekly at time 0, 2 and 4 weeks. It was then given every 3 weeks at weeks 7, 10 and 13. CYC dose was calculated using the table below. Individual CYC doses did not exceed 1200mg. Mesna – as prophylaxis against haemorrhagic cystitis – was administered alongside the CYC. The dose was 40% that of the pulse of CYC (in mg).

| **Age (years)** | **Estimated GFR (ml/min)**  **>30** | **Estimated GFR (ml/min)**  **≤30** |
| --- | --- | --- |
| < 60 | 15.0mg/kg/pulse | 12.5mg/kg/pulse |
| > 60 & < 70 | 12.5mg/kg/pulse | 10.0mg/kg/pulse |
| > 70 | 10.0mg/kg/pulse | 7.5mg/kg/pulse |

*Prophylactic treatment*

All patients were prescribed gastric protection with either lansoprazole 30mg daily or ranitidine 150mg b.d. Infection prophylaxis was with cotrimoxazole 480mg daily. Protection against bone loss was with a bisphosphonate if the estimated GFR was >35 ml/min together with oral calcium supplementation. If the estimated GFR was <35 ml/min, patients were prescribed a combination of activated vitamin D and supplemental calcium.

**Supplementary Table 1**

| **Characteristic** | **CYC**  **(n=68)** | **Rituximab**  **(n=22)** | **p value** |
| --- | --- | --- | --- |
| **Number of patients entering remission (%)**  **Mean time to remission (days)**  **Median time to remission (days & IQR)**  **Number of patients relapsing (%)**  **Mean time to relapse (days)**  **Median time to relapse (days & IQR)** | 67 (99)  88 ± 8  76 (42)  12 (18)  1034 ± 106  795 (1378) | 20 (91)*  155 ± 81  80 (39)  2 (9)  1129 ± 190  927 (850) | 0.42  0.16  0.82  0.50  0.66  0.43 |

* 1 patient died in this group before entering remission

**Supplementary Table 2**

| **Characteristic** | **<70 years**  **(n=75)** | **≥70 years**  **(n=29)** | **p value** |
| --- | --- | --- | --- |
| **Number receiving rituximab (%)**  **Cumulative glucocorticoid dose at 3 months (g)**  **Number of patients achieving remission (%)**  **Number of patients relapsing (%)**  **12 months survival (%)** | 8 (11)  2.33 ± 0.30  74 (99)  14 (19)  75 (100) | 14 (48)  2.39 ± 0.35  27 (93)*  6 (24)  27 (93) | **0.0001**  0.39  0.19  0.79  0.41 |

* 2 patients died in this group before entering remission
